# Unraveling the Enigma: A Case Report on Unilateral Ovarian Dermoid Cyst

**DOI:** 10.7759/cureus.53700

**Published:** 2024-02-06

**Authors:** H S Deeksha, Sandhya Pajai, Dharmesh J Patel, Vinayak U Navalihiremath, Garapati Jyotsna

**Affiliations:** 1 Obstetrics and Gynecology, Jawaharlal Nehru Medical College, Datta Meghe Institute of Higher Education and Research, Wardha, IND; 2 Internal Medicine, Aarupadai Veedu Medical College, Pondicherry, IND

**Keywords:** gynecology, unilateral, teraton, teratoma, dermoid ovarian cyst

## Abstract

Mature teratomas, also known as ovarian dermoid cysts, are benign embryonal tumors that develop slowly. One of the following imaging techniques is commonly employed to evaluate these cysts: transvaginal ultrasound, pelvic ultrasonography, magnetic resonance imaging, or computed tomography. The two surgical methods most frequently used for removing persistent or very large cysts are laparoscopy and laparotomy. A 42-year-old female, who is P3L1D2 with a history of previous cesarean section, presented with an abdominal mass that had been gradually increasing in size over the past five months. She also reported lower abdominal pain for the last five days. Upon further evaluation, she was diagnosed with a left ovarian dermoid cyst. The patient underwent exploratory laparotomy, during which a total abdominal hysterectomy with bilateral salpingo-oophorectomy was performed. The cut section of the gross cyst specimen revealed abundant sebaceous fluid and a large tuft of hair, which was confirmed by histopathology. The patient was followed up every three months for a year. Ovarian tumors typically manifest with nonspecific symptoms. The early recognition of dermoid cysts and prompt intervention are crucial to prevent potential complications.

## Introduction

Mature cystic teratomas (MCT), commonly referred to as ovarian dermoid cysts, stand as prevalent benign ovarian tumors affecting both adult females and adolescents. Their frequency in the realm of benign masses is noteworthy, constituting 70% before menopause and decreasing to 20% after menopause [1-3]. These tumors are distinguished by their slow growth and benign embryonal nature, marking them as the most prevalent germinative ovarian tumors in females of reproductive age. Their origin can be traced back to the entrapment of ectodermal components during closure processes [4]. The composition of mature cystic teratomas encompasses adult ecto-/endo-/mesodermal tissue, encompassing diverse elements such as the teeth, skin, fat, hair, muscle, and even brain and thyroid tissues [2-5]. When the predominant tissue in MCT is ectodermal in nature, the term "dermoid cyst" is used to describe them.

Dermoid cysts, categorized as benign germ cell tumors, contribute to 10%-25% of all ovarian tumors, and bilateral involvement is observed in approximately 10%-15% of cases. The malignant transformation of mature components is rare, transpiring in only 1%-2% of dermoid cysts. Squamous cell carcinoma takes precedence in 80%-90% of these instances, with adenocarcinoma being the most prevalent subtype. The likelihood of malignant transformation is heightened in postmenopausal females [6,7]. This comprehensive understanding sheds light on the multifaceted nature of mature cystic teratomas and their clinical implications, emphasizing their prevalence, composition, and the rare potential for malignant transformation.

## Case presentation

A 42-year-old female, who is P3L1D2 with a history of a previous cesarean section, presented to the outpatient gynecological clinic in Sawangi, Wardha. She reported a five-month history of an abdominal mass that had gradually increased in size, accompanied by lower abdominal pain for the past five days. The patient also mentioned experiencing early satiety, constipation, and difficulty in passing stools. There were no complaints of abdominal bloating, vomiting, weight loss, intermenstrual bleeding, leg swelling, or lower limb varicosity. Her medical and surgical history was unremarkable, and the general examination revealed normal findings with stable vital signs.

A suprapubic bulge moving with respiration was noted during the abdominal examination, and the abdomen appeared full. The umbilicus was centrally located, and a visible scar from the previous lower-segment cesarean section (LSCS) was present. No evidence of dilated veins was observed. Palpation revealed a mass of 18 weeks in size, with the lower boundary not palpable and the upper and lateral boundaries well-defined. The mass exhibited mobility from side to side and was not fixed to the skin's surface or any underlying structures. No audible bruits were over the mass, and ascites were not appreciable. Bowel sounds were normal.

The examination of the cervix and vaginal region using a speculum revealed no abnormalities, but a mass separate from the uterus was discovered during a vaginal examination. Perrectal examination yielded normal findings. The patient's blood, serum, and tumor markers, including cancer antigen (CA) 125 and carcinoembryonic antigen (CEA), were within normal ranges. CA 19.9 was at 45 U/mL, and serum beta-human chorionic gonadotropin (HCG) was measured at 32.31 mIU/mL (Table 1).

**Table 1:** Laboratory Results CA: cancer antigen

Serial number	Name of investigation	Patient value	Reference value
1	Serum beta-human chorionic gonadotropin (bHCG)	32.3 mIU/mL	<5 mIU/mL
2	Carcinoembryonic antigen (CEA)	2 ng/mL	<2.5 ng/mL
3	CA 19.9	45 U/mL	<37 U/mL
4	CA 125	38 U/mL	<35 U/mL

**Figure 1:**
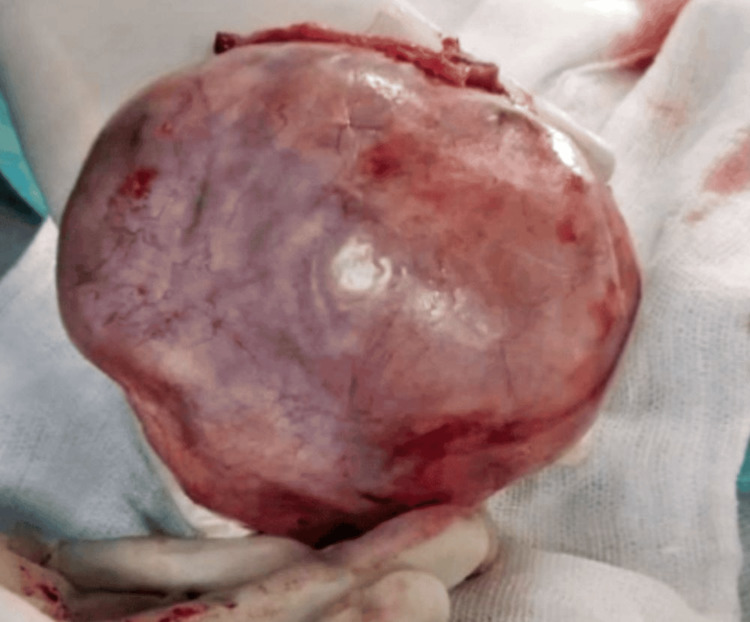
Dermoid Cyst With Intact Capsule

**Figure 2:**
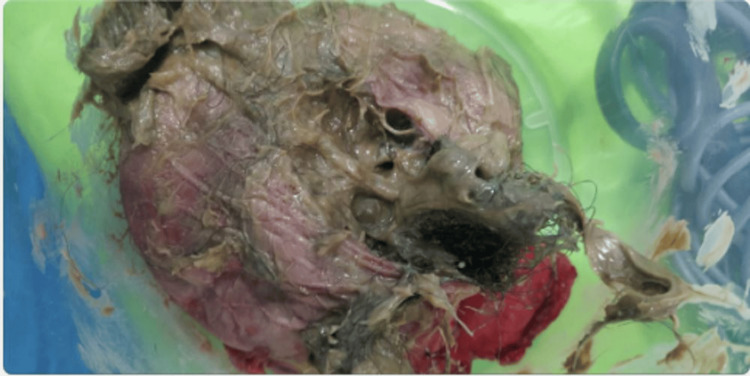
Cut Open Section of Dermoid Cyst Greasy Yellow Material From Sebaceous Gland, Fat, and Skin Adnexal Structures, Hair

The ultrasound examination revealed a normal right ovary measuring 3.2×2.9 cm. In contrast, the left ovary exhibited a well-defined cystic mass lesion measuring 10.6×7.2×9.2 cm in the pelvis, predominantly arising from the left ovary. This lesion displayed septations and an echogenic solid component, leading to a probable diagnosis of either a dermoid cyst or a hemorrhagic cyst. The diagnosis was determined to be an ovarian cyst on the left side. The patient received counseling regarding the necessity of an exploratory laparotomy, with the potential risk of losing the ovaries. Written informed consent was obtained, and the patient underwent surgery under anesthesia with the physician's consent. Intraoperatively, a cystic ovarian mass measuring 16×12 cm with solid components and visible hair through the transparent wall was observed. The mass was not adherent to other organs, and there was no evidence of torsion. The surgical procedure performed was a total abdominal hysterectomy with bilateral salpingo-oophorectomy, with careful measures taken to prevent cyst rupture. A histopathological examination of the tissues was conducted. The patient was discharged seven days post operation following the surgery, with sutures removed. A follow-up visit to the gynecological clinic occurred four weeks later. A gross examination of the specimen revealed an assemblage of solidified material packed with a hair tuft lodged within a paper-thin wall (Figure 1). The reported cystic ovarian mass exhibited a cavity lined with mature epidermis and included the adnexa of the skin (Figure 2).

The histopathology reports revealed cystic lesions with multiple septations characterized by stratified squamous epithelia. The cystic wall exhibited the presence of skin appendages. A benign cystic teratoma was identified, and the findings were consistent with this diagnosis. Following the surgical intervention, the patient underwent regular follow-up for one year, with assessments conducted every third month. During this period, the patient reported no abdominal pain or swelling.

## Discussion

An alternative term for a dermoid cyst is a benign cystic teratoma, with the term "teratoma" originating from the Greek word "teraton," meaning monster. The term "dermoid cyst" was first coined by Leblanc in 1831. These cysts, classified as benign germ cell tumors, constitute 10%-25% of all ovarian cancers [8-10]. Dermoid cysts develop when ectodermal components become trapped along the lines of embryonic closure, resulting in benign cutaneous developmental anomalies [11]. The discovery of ovarian cysts often causes significant anxiety in females due to concerns about malignancy and infertility. Dermoid cysts are typically benign and unilateral, though occasional bilateral occurrences can happen. Most cysts are asymptomatic, but in some cases, they may cause severe stomach pain, discomfort, bloating, anorexia, or other signs of gastrointestinal disturbance [9].

These cysts commonly occur between the ages of 25 and 45, with 10%-15% being bilateral and 85%-90% unilateral. Small dermoid cysts are often asymptomatic, becoming symptomatic with increased size [12]. The complications associated with dermoid cysts include infection, torsion, hemorrhage, and rupture, with 3%-4% recurrence, and rare malignant transformation. Chronic granulomatous peritonitis can result from spillage during operative procedures [12]. Squamous cell carcinoma accounts for approximately 90% of mature teratomas and 1%-2% of dermoid cyst malignancies, with infection-related cases only 1% [4]. Ultrasound is a highly useful noninvasive method for diagnosing ovarian cysts, with magnetic resonance imaging or computed tomography scans being alternative options. Diagnosis confirmation is achieved through histology. Clinical signs often involve the size, compression, or torsion of the slow-growing lesion and chemical peritonitis from an intra-abdominal leak of cholesterol material [13]. Treatment depends on the size and symptoms of the cyst. Symptomatic large cysts are usually removed via laparotomy/laparoscopy, while asymptomatic cysts often resolve independently.

Laparoscopy remains the preferred method for treating ovarian dermoid cysts due to its less invasive nature; however, it increases the risk of cyst content leakage and peritonitis. For young females desiring pregnancy, laparoscopic treatment is a safer and preferred option, offering unilateral/bilateral salpingo-ovariectomy cystectomy. Using a laparoscopic endobag during treatment has shown promising results, reducing operating time and preventing potential leakage [14]. Laparoscopy offers several advantages, including a decreased risk of wound complications, reduced ileus and discomfort, less postsurgical pain, shorter hospital stays, lower adhesion development, and an earlier return to regular activities.

## Conclusions

In conclusion, ovarian tumors often manifest with symptoms that lack specificity, making early detection and intervention vital for optimal outcomes. Research indicates a notable association between the size of the cyst and the overall survival prognosis. Recognizing dermoid cysts at an early stage is particularly crucial to preempt potential complications. This underscores the importance of vigilant monitoring, timely diagnosis, and proactive intervention strategies in the management of ovarian tumors, with the ultimate goal of improving patient outcomes and mitigating adverse consequences.
